# From Byproduct to Regulator: The Expanding Role of Lactate and Lactylation in Cardiovascular Physiology and Disease

**DOI:** 10.3390/biology15080642

**Published:** 2026-04-18

**Authors:** Hanqiang Deng

**Affiliations:** 1 Yale Cardiovascular Research Center, Yale University School of Medicine, New Haven, CT 06511, USA; hanqiang.deng@yale.edu; 2 Section of Cardiovascular Medicine, Department of Internal Medicine, Yale University School of Medicine, New Haven, CT 06511, USA

**Keywords:** lactate, lactylation, glycolysis, endothelial cells, Warburg effect, lactate shuttle theory, post-translational modification, cardiovascular diseases

## Abstract

Metabolic reprogramming plays a critical role in the pathogenesis of cardiovascular diseases. Historically regarded as a metabolic waste product, lactate has recently emerged as a critical regulator of vascular biology, exerting both metabolic and signaling functions. Moreover, the discovery of protein lactylation, a novel post-translational modification derived from lactate, has revealed a direct link between metabolic flux and gene regulation. This review provides a comprehensive overview of the evolving roles of lactate and lactylation in cardiovascular physiology and disease, offering insights into potential therapeutic interventions. It first provides a historical perspective of lactate and lactylation, followed by an overview of lactate metabolism, lactate shuttle theory and signaling pathways. It then discusses the mechanism and regulation of lactylation, focusing on its writers, erasers, and readers. Finally, this review summarizes clinical implications of lactate and lactylation in various cardiovascular diseases, including atherosclerosis, pulmonary hypertension, myocardial infarction, heart failure, and diabetic vascular complications. A deeper understanding of the mechanisms underlying the lactate–lactylation axis may facilitate the development of new therapeutic strategies to prevent or treat cardiovascular diseases.

## Simple Summary

This review explores the evolving understanding of lactate, once dismissed as a simple metabolic waste product, and its newly discovered role as a key regulator in cardiovascular health and disease. The article traces the historical journey of lactate research, from its initial discovery to the groundbreaking finding that lactate can drive protein lactylation, a novel post-translational modification that directly links cellular metabolism to gene expression. This review examines how lactate functions both as an energy source and signaling molecule, while lactylation serves as an epigenetic mechanism influencing chromatin structure and transcriptional activity. Rapidly growing evidence indicates that the lactate–lactylation axis contributes to the development of various cardiovascular diseases. By summarizing current understanding of these mechanisms, this review suggests that targeting lactate metabolism and lactylation pathways may open new therapeutic avenues for preventing or treating cardiovascular diseases.

## 1. Introduction

Cardiovascular disease (CVD) remains the leading cause of morbidity and mortality worldwide, encompassing a broad spectrum of disorders including atherosclerosis, hypertension, myocardial infarction, and heart failure [1]. The pathogenesis of CVD is multifactorial and involves a complex interplay among metabolic dysregulation, chronic inflammation, and biomechanical forces within the vascular system [2,3,4]. Central to this interplay is the profound metabolic reprogramming of endothelial cells (ECs), characterized by shifts in glycolytic flux, mitochondrial respiration, and substrate utilization [5]. These metabolic adaptations not only meet altered energetic demands but also actively regulate inflammatory signaling, endothelial plasticity, and pathological vascular remodeling [6,7,8].

Glycolysis is a fundamental metabolic pathway that serves as the primary process for glucose breakdown to generate adenosine triphosphate (ATP) [9]. This cytosolic process comprises ten enzymatic steps that convert one molecule of glucose into two molecules of pyruvate, along with a net gain of two ATP and two nicotinamide adenine dinucleotide (NADH) molecules. Glycolysis is highly conserved across species and functions under both aerobic and anaerobic conditions. Under aerobic conditions, pyruvate enters the mitochondria for further oxidation via the tricarboxylic acid (TCA) cycle, whereas in anaerobic conditions, pyruvate is converted to lactate through lactate dehydrogenase (LDH) [10].

Lactate, historically viewed as a metabolic waste byproduct, has emerged as an important metabolic fuel and signaling molecule [11]. It plays critical roles in diverse physiological processes, including the regulation of gene expression, immune responses, and angiogenesis [12]. Lactate can be utilized as an energy substrate by the heart, liver, and brain or serve as a precursor for gluconeogenesis in the liver. Its dynamic production and clearance are tightly controlled, with elevated levels often associated with pathological conditions such as sepsis, cancer, metabolic disorders, and cardiovascular diseases [13].

Lactylation is a recently discovered post-translational modification (PTM) of lysine residues, which involves the addition of a lactyl group derived from lactate [14]. This modification links cellular metabolism to epigenetic regulation, highlighting the emerging role of metabolic intermediates in controlling gene expression and cellular functions. First identified in histones, lactylation has been shown to influence chromatin structure and transcriptional activity, particularly in response to metabolic shifts such as increased aerobic glycolysis and lactate production. For instance, histone lactylation has been implicated in promoting the expression of genes associated with wound healing, immune responses, and tumor progression. As a novel regulatory mechanism, lactylation provides insight into how metabolic states can dynamically influence cell fate and function. This makes it a promising area of research for understanding disease pathogenesis and developing therapeutic interventions.

Within the cardiovascular system, accumulating evidence suggests that the lactate–lactylation axis contributes to vascular inflammation, endothelial dysfunction, immune responses, and cardiac remodeling [15,16,17]. These findings highlight an emerging paradigm in which metabolic intermediates function not only as substrates for energy production but also as regulators of epigenetic and transcriptional networks.

This review provides a comprehensive synthesis of current knowledge regarding lactate and lactylation in cardiovascular biology. It first traces their historical discovery, elucidates their metabolic and signaling roles, and then explores mechanistic links to vascular physiology and disease. Lastly, this review discusses clinical implications of lactate and lactylation in cardiovascular diseases, such as atherosclerosis, pulmonary hypertension, myocardial infarction, heart failure, and diabetic vascular complications. We do so with the hope that an improved understanding of the mechanisms can accelerate the development of new therapies to prevent or treat cardiovascular diseases.

## 2. Historical Perspective

**Figure 1:**
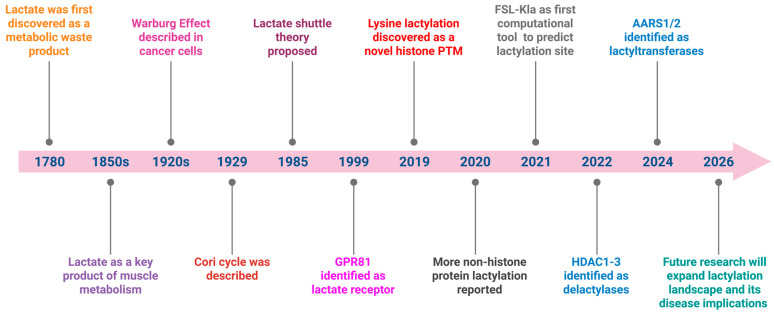
Historical timeline of key discoveries in lactate and lactylation research. In 1780, lactate was first discovered in sour milk. In the 1850s, lactate was identified as a key product of muscle metabolism. In the 1920s, the Warburg effect was discovered by Otto Warburg. In 1929, Carl and Gerty Cori further advanced the understanding of lactate metabolism by describing the Cori cycle. In 1985, the lactate shuttle theory was first proposed by George A. Brooks. In 2019, lactylation was first identified as a new histone post-translational modification. Subsequent studies in 2020 expanded lactylation to non-histone proteins. In 2021, FSL-Kla, a few-shot learning-based hybrid system, was reported for lactylation site prediction. In 2022, HDAC1-3 were identified as delactylases, and in 2024, AARS1/2 were reported as lactyltransferases. Looking ahead to 2026 and beyond, research will continue to expand the lactylation landscape and its implications in human diseases. Created with BioRender.com.

The history of lactate can be traced back to the late 18th century, when Swedish chemist Carl Wilhelm Scheele first discovered it in sour milk in 1780, naming it after its source (Figure 1) [13]. In the mid-19th century, Johannes Wislicenus identified lactate as a key product of muscle metabolism, linking it to muscle activity and fatigue [18]. For much of the early 20th century, lactate was widely regarded as a dead-end waste product responsible for muscle fatigue and acidosis, a view cemented by A.V. Hill and colleagues via the “oxygen debt” hypothesis [19]. In 1929, Carl and Gerty Cori further advanced the understanding of lactate metabolism by describing the Cori cycle. In this pathway, lactate produced by skeletal muscle is transported to the liver and converted back into glucose through gluconeogenesis [20]. This process links peripheral energy metabolism with hepatic glucose production. However, this traditional perception began to shift in the late 20th century with groundbreaking work by George A. Brooks, who demonstrated that lactate is not merely a byproduct but an essential metabolic fuel and signaling molecule [21]. The “lactate shuttle” hypothesis introduced the concept of lactate as a dynamic intermediary in energy metabolism, capable of being transported between tissues and organs. This evolving perspective highlights lactate’s pivotal role in health and disease, transforming it from a simple waste product to a key player in metabolic and cellular biology.

The history of lactylation is relatively recent, emerging as a groundbreaking discovery in the field of epigenetics and metabolism. Lactylation was first identified in 2019 by a research team led by Yingming Zhao, who discovered that lactate could serve as a donor for a novel post-translational modification of lysine residues on histones (Figure 1) [22]. This modification, termed histone lactylation, revealed a direct link between cellular metabolism and gene regulation. Initially observed in macrophages during inflammatory responses, lactylation was shown to regulate gene expression programs involved in tissue repair and immune function. This discovery reshaped our understanding of lactate, previously thought to be merely a metabolic byproduct, by highlighting its role in influencing chromatin dynamics and cellular function. Since its identification, lactylation has been implicated in a growing range of physiological and pathological processes, including cancer progression, immune modulation, cardiovascular and metabolic diseases [23]. This makes it a promising area of research for uncovering novel therapeutic targets.

Together, these historical advances chart the evolution of lactate’s scientific identity from waste product to metabolic shuttle, signaling mediator, and epigenetic regulator. This trajectory underscores a broader shift in biomedical research: the recognition that intermediary metabolites are not merely passive participants in metabolism but active determinants of cell fate and tissue function. Lactate and lactylation now stand at the intersection of metabolism, signaling, and epigenetics, offering new perspectives on cardiovascular health and disease.

## 3. Lactate Metabolism and Signaling

### 3.1. Lactate Metabolism

**Figure 2:**
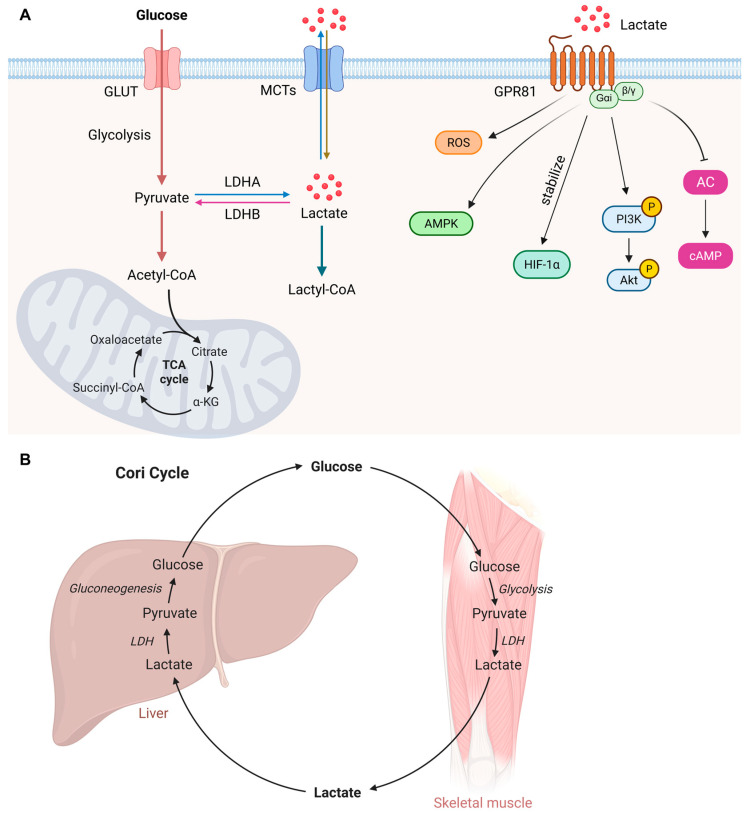
Lactate metabolism and signaling. (**A**) Lactate is generated during glycolysis when glucose is metabolized to pyruvate in the cytoplasm. Under conditions of high glycolytic flux or limited mitochondrial oxidation, pyruvate is converted to lactate via lactate dehydrogenase (LDH), a reaction that simultaneously regenerates nicotinamide adenine dinucleotide (NAD^+^) to sustain continued glycolytic ATP production. Lactate is transported across cell membranes by monocarboxylate transporters (MCTs), functioning as a metabolic fuel in the “lactate shuttle”. Concurrently, it acts as an extracellular signaling molecule by activating the G protein-coupled receptor 81 (GPR81), which influences multiple intracellular signaling cascades. (**B**) Cori cycle, or lactic acid cycle, is a crucial metabolic pathway where lactate produced by anaerobic glycolysis in peripheral tissues (e.g., skeletal muscles) is transported via the blood to the liver. The liver converts this lactate back into glucose through gluconeogenesis. GLUT, glucose transporter. AC, adenylyl cyclase. Created with BioRender.com.

Lactate metabolism is a critical component of cellular energy homeostasis and an essential driver of metabolic flexibility across tissues [13]. Lactate is primarily generated during glycolysis, in which glucose is metabolized to pyruvate in the cytoplasm. Under conditions of high glycolytic flux (aerobic glycolysis) or limited mitochondrial oxidation (anaerobic glycolysis), pyruvate is converted to lactate via lactate dehydrogenase (LDH) (Figure 2A). This reaction simultaneously regenerates nicotinamide adenine dinucleotide (NAD^+^) to sustain continued glycolytic ATP production. Importantly, lactate production is not restricted to anaerobic conditions. Even in the presence of oxygen, many cells, especially endothelial cells, immune cells, and cancer cells, prefer aerobic glycolysis for energy generation and biosynthetic needs. This aerobic glycolysis, known as the “Warburg effect”, results in substantial lactate accumulation [24].

Importantly, the functional significance of aerobic glycolysis differs between physiological and pathological contexts in the cardiovascular system [25]. In healthy ECs, glycolysis predominates even under normoxic conditions, supporting rapid ATP production, biosynthetic processes, and key functions such as proliferation, migration, angiogenesis, and vascular homeostasis, while preserving mitochondrial oxygen [26]. This process is tightly coupled to lactate export and utilization by adjacent oxidative tissues, maintaining metabolic balance. In contrast, under pathological conditions such as atherosclerosis, pulmonary hypertension, and heart failure, sustained glycolytic reprogramming becomes maladaptive, leading to excessive lactate accumulation, redox imbalance, and intracellular acidification [25,27]. These changes drive pro-inflammatory signaling, endothelial-to-mesenchymal transition (EndMT), vascular smooth muscle cells (VSMCs) proliferation, and extracellular matrix remodeling. Thus, the “Warburg-like” phenotype in cardiovascular disease reflects a dysregulated amplification of an otherwise physiological metabolic program.

Once produced, lactate serves as a key metabolic fuel that can be transported between tissues via monocarboxylate transporters (MCTs). In the Cori cycle, lactate is shuttled from peripheral tissues, like skeletal muscle, to the liver, where it is converted back to glucose via gluconeogenesis (Figure 2B) [28]. Lactate can also be oxidized directly in tissues such as the heart, brain, and oxidative muscle fibers to generate energy through the tricarboxylic acid (TCA) cycle. Beyond its role as a metabolic substrate, lactate acts as a signaling molecule, influencing processes such as angiogenesis, immune modulation, and epigenetic regulation via histone lactylation. Dysregulation of lactate metabolism is linked to various pathological conditions, including cancer, metabolic disorders, and cardiovascular diseases.

### 3.2. Lactate Shuttle Theory

The lactate shuttle theory, first proposed by George A. Brooks in 1985 [21], revolutionized our understanding of lactate as a central intermediary in energy production and transport, rather than merely a metabolic byproduct. According to this theory, lactate acts as a key energy substrate that is produced in glycolytic tissues (e.g., exercising muscle during exercise) and transported via monocarboxylate transporters (MCTs) to oxidative tissues (e.g., heart, brain, liver), where it can be metabolized for energy or used in gluconeogenesis [29].

There are three major types of lactate shuttles: (1) cell-to-cell shuttles, where lactate is transported from glycolytic cells to oxidative cells for oxidation [30]. In the vascular context, ECs can export lactate via MCT4, which is then taken up by adjacent VSMCs or perivascular cells expressing MCT1; (2) intracellular shuttles, where lactate moves between cytosolic and mitochondrial compartments within the same cell to fuel mitochondrial respiration [31,32]. Within oxidative muscle cells or cardiomyocytes, lactate can be transported into mitochondria via mitochondrial MCT1 and converted to pyruvate by mitochondrial LDH for entry into the TCA cycle; (3) organ-level shuttles, such as between skeletal muscle, liver, heart, and brain [33]. A classic example is the Cori cycle, where lactate released from exercising muscle is transported via the bloodstream to the liver for gluconeogenesis. The concept also highlights the role of lactate in intracellular signaling and its function as a metabolic bridge between glycolytic and oxidative cells. For example, within muscle cells, lactate can shuttle between different muscle fibers or even within compartments of the same cell to optimize energy usage. The lactate shuttle theory not only redefined lactate as a vital component of systemic energy homeostasis but also provided a framework for understanding its involvement in physiological processes such as exercise adaptation, tissue repair, and brain metabolism.

### 3.3. Lactate Signaling

Beyond its metabolic functions, lactate acts as a signaling molecule that modulates gene expression, immune responses, vascular function, and cell fate decisions [15,34,35]. Lactate activates the G-protein-coupled receptor GPR81 (also known as HCAR1) [36], which is expressed in adipocytes, muscle cells, and vascular cells. GPR81 activation inhibits adenylyl cyclase (AC), reducing cAMP levels and modulating downstream signaling pathways [37]. Lactate influences multiple intracellular signaling cascades (Figure 2A). It activates adenosine monophosphate (AMP)-activated protein kinase (AMPK), a master metabolic sensor that regulates endothelial homeostasis, nitric oxide (NO) production, and autophagy [38,39]. Lactate also stabilizes hypoxia-inducible factor-1 alpha (HIF-1α) under normoxic conditions, promoting expression of glycolytic genes and angiogenic factors [40]. In ischemic heart disease, lactate-induced HIF-1α stabilization contributes to collateral vessel formation following myocardial infarction [40,41]. However, sustained HIF-1α activation in VSMCs also drives pathological vascular remodeling [42,43]. Additionally, lactate can modulate reactive oxygen species (ROS) production and redox signaling through NADH/NAD^+^ ratio alterations [44,45]. Furthermore, GPR81 signaling has been implicated in the regulation of vascular tone and angiogenesis, partly through activation of the PI3K/Akt pathway [46,47]. Lactate-GPR81 signaling may influence endothelin-1 production, thereby contributing to vasoconstriction and vascular dysfunction [48,49]. These signaling cascades regulate processes such as angiogenesis, immune responses, and cellular adaptation to hypoxia, making lactate a critical player in tissue homeostasis and disease states. Understanding lactate signaling provides insights into its role as a metabolic and signaling hub, linking energy metabolism to cellular function in health and disease.

## 4. Lactylation: Mechanism and Regulation

### 4.1. Mechanism of Lactylation

**Figure 3:**
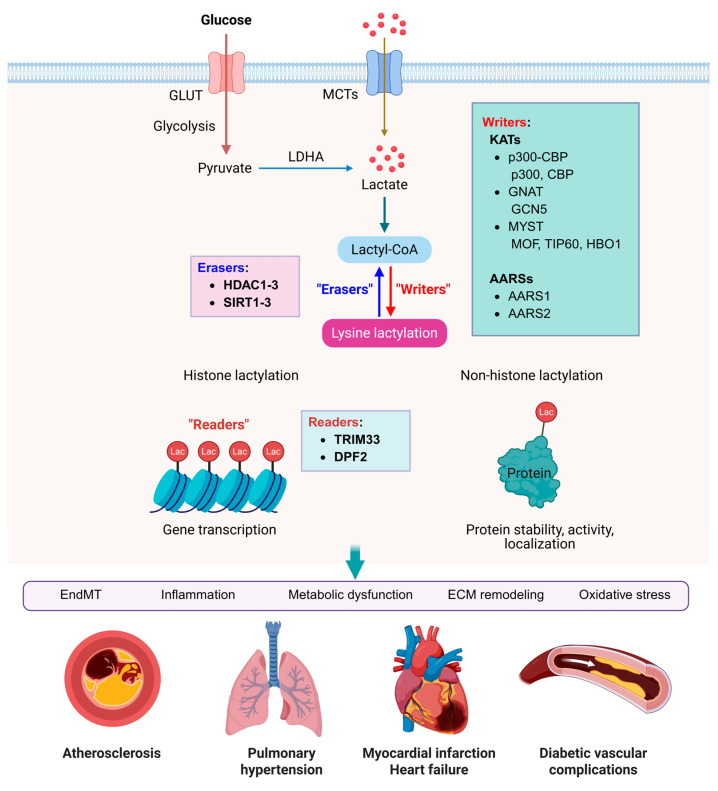
Regulatory mechanisms of lactylation and its implications in cardiovascular diseases. Lactate generated through glycolysis, or imported from the extracellular environment, can be converted to lactoyl-coenzyme A (lactoyl-CoA), which serves as a key donor substrate for protein lactylation. During this process, the lactoyl group is transferred to lysine residues on target proteins by enzymatic “writers,” whereas “eraser” enzymes remove the modification. In addition, “reader” proteins recognize lactylated lysine residues and translate these epigenetic marks into downstream signaling events that regulate diverse biological processes, including inflammation, EndMT, metabolic dysfunction, ECM remodeling, and oxidative stress. Histone lactylation regulates gene transcription by altering chromatin, while non-histone lactylation primarily modifies functional proteins via regulating protein stability, activity, localization, and associated signaling pathways. Those processes finally contribute to various cardiovascular diseases, such as atherosclerosis, pulmonary hypertension, myocardial infarction, heart failure and diabetic vascular complications. Created with BioRender.com.

Lactylation involves the covalent addition of a lactyl group to the ε-amino group of lysine residues, utilizing lactyl-coenzyme A (lactyl-CoA) as the donor. Histone lactylation has been identified on multiple lysine residues, including H3K18, H3K23, H4K8, and H4K12, where it is generally associated with transcriptional activation [50,51,52]. Like other common types of PTMs, lactylation is dynamically regulated by two classes of enzymes: ‘writers’, which catalyse the addition of the modification, and ‘erasers’, which facilitate its removal (Figure 3) [53].

### 4.2. Writers: Lactyltransferases

Lactyltransferase is an enzyme that facilitates the transfer of lactyl groups to specific target molecules. So far, lactylation writers can be broadly classified into two categories based on their catalytic mechanisms: acyltransferases, which utilize lactyl-CoA as the acyl donor, and alanyl-tRNA synthetases (AARSs), which use lactate and ATP to transfer lactyl groups onto lysine residues.

Lysine acyltransferases (KATs), originally characterized as enzymes that catalyze lysine acetylation using acetyl-CoA, are classified into three major families based on sequence homology and structural features [54]: the GCN5-related N-acetyltransferase (GNAT) family, the p300/CBP family, and the MYST family, which includes monocytic leukemia zinc finger (MOZ), yeast binding factor 2 (YBF2; also known as Sas3), Sas2, and tat-interactive protein 60 (TIP60) [55]. Subsequent studies have further expanded the repertoire of lactylation-competent enzymes, showing that KATs across all three families possess lactyltransferase activity. These include KAT2A (GCN5) [56,57], KAT3A (CBP) [58], KAT5 (TIP60) [59], KAT7 (HBO1) [60] and KAT8 (MOF) [61], suggesting that lysine lactylation may be a broadly regulated epigenetic modification mediated by multiple members of the KAT family. In addition, Liu et al. have demonstrated that p300 interacts with guanosine triphosphate (GTP)-specific succinyl-CoA synthetase (GTPSCS) in the nucleus to form a lactyltransferase complex, promoting histone lactylation [62].

Recently, a novel class of lactylation writer proteins, known as alanyl-tRNA synthetases (AARSs), was identified [57,63,64]. AARSs, including AARS1 and AARS2, encode class II aminoacyl-tRNA synthetases responsible for catalyzing the attachment of L-alanine to tRNA during protein translation. Notably, they can acquire additional functionality under specific metabolic or inflammatory conditions. AARS1 functions predominantly in the cytoplasm, whereas AARS2 is primarily localized to the mitochondria. The mitochondrial isoform AARS2 was first identified as an enzyme catalysing lactylation using lactate and ATP [65]. Subsequently, the cytosolic isoform AARS1 was also shown to exhibit lactyltransferase activity [64].

### 4.3. Erasers: Delactylases

Delactylation is a recently discovered enzymatic process that removes lactyl groups from lysine residues on histones and potentially other proteins, serving as a regulatory mechanism to reverse the effects of lactylation. The balance between lactylation and delactylation allows cells to rapidly respond to metabolic changes. While the specific enzymes responsible for delactylation are still under investigation, emerging evidence suggests that delactylation plays a crucial role in fine-tuning gene expression and maintaining cellular homeostasis. Class I histone deacetylases (HDAC1-3) [66], certain sirtuins (SIRT1-3) [67] have been implicated in delactylation, although substrate specificity and in vivo relevance remain incompletely defined (Figure 3). For instance, delactylation may act as a counterbalance to histone lactylation, regulating inflammatory responses, metabolic reprogramming, and tumor progression. Identifying delactylation enzymes could advance our understanding of metabolism-epigenetics interplay, especially in cancer, cardiovascular disease, and immune dysfunction, where lactate signaling is dysregulated.

### 4.4. Specificity Mechanisms

Although an increasing number of lactyltransferases and delactylases have been identified, the enzymatic landscape remains incompletely defined. An important unresolved question is how lactyltransferases, including p300/CBP and other KATs, discriminate between lactyl-CoA and acetyl-CoA as acyl donors. Current evidence suggests that substrate selection is governed by multiple factors rather than strict specificity [68]. Intracellular metabolite availability plays a central role, as elevated glycolytic flux increases lactate and lactyl-CoA pools, shifting the balance toward lactylation [69]. Structural flexibility within lysine KATs’ active sites enables accommodation of different acyl groups with modest differences in binding affinity, supporting catalytic promiscuity. Subcellular compartmentalization further refines this process, with localized lactyl-CoA production favoring site-specific histone lactylation. Additionally, alternative pathways such as AARS1/2-mediated lactylation can bypass lactyl-CoA entirely, highlighting mechanistic diversity [70]. In parallel, delactylases such as HDACs and SIRTs regulate the removal of lactyl groups and exhibit differential catalytic efficiencies toward distinct acyl modifications. The larger, more polar lactyl group often slows diacylation compared to acetylation. In the cardiovascular microenvironment, cues such as hypoxia, shear stress, and inflammation dynamically regulate enzyme activity and metabolite pools, collectively shaping the balance between acetylation and lactylation. However, the precise biochemical determinants of acyl-group specificity remain incompletely understood and warrant further investigation.

### 4.5. Readers

Readers are effector proteins that specifically recognize and bind to histones in a PTM-specific manner (Figure 3). Smith et al. identified the TRIM33 PHD-bromodomain as the only one capable of binding histone peptides bearing lactylation [71]. In addition, double PHD fingers 2 (DPF2) has been identified as a histone H3K14la reader that regulates gene transcription [72]. However, research on lactylation-specific reader proteins remains limited, and further studies are needed to identify additional readers and clarify their molecular functions.

### 4.6. Translational Considerations and Challenges

Despite rapid progress in identifying enzymes involved in lactylation, several critical challenges remain regarding their validation, feasibility, and therapeutic potential [73]. First, although multiple candidate “writer” enzymes have been proposed, their roles as bona fide lactyltransferases are not yet fully validated in vivo. Most current evidence is derived from in vitro biochemical assays or cell-based studies, and each enzyme’s contribution under physiological or pathological conditions remains unclear. Moreover, it remains unclear whether these enzymes function as dedicated lactyltransferases or exhibit catalytic promiscuity driven by intracellular metabolite availability. Second, the feasibility of therapeutically targeting lactylation pathways remains uncertain. While modulation of upstream metabolic processes, such as glycolysis or lactate production, has shown promise in preclinical models, direct targeting of lactyltransferases or delactylases is still in its early stages. A major limitation is the lack of selective small-molecule modulators that specifically regulate lactylation without affecting acetylation. Finally, potential side effects must be carefully considered. Given the central role of lactate metabolism in energy homeostasis, immune regulation, and tissue repair, interventions targeting the lactate–lactylation axis may induce side effects. For example, inhibition of lactylation could impair physiological processes such as wound healing, angiogenesis, and immune resolution. Thus, rigorous in vivo validation, better understanding of enzyme specificity, and more selective pharmacological tools are needed before clinical translation of lactate–lactylation targeting.

## 5. Lactate and Lactylation in Cardiovascular Diseases

### 5.1. Atherosclerosis

Atherosclerosis (AS) is a chronic inflammatory disease of the arterial wall involving multiple cell types, including vascular ECs, vascular smooth muscle cells (VSMCs), and monocyte-derived macrophages [74]. Metabolic reprogramming, particularly enhanced aerobic glycolysis, has emerged as a key regulator of cellular activation and inflammatory responses during atherogenesis [75]. Notably, aerobic glycolysis is more prominently engaged than anaerobic glycolysis in atherosclerotic lesions, supporting energy production, biosynthesis, and signaling processes that drive vascular inflammation and plaque progression [76]. In a community-based study on AS risk, carotid magnetic resonance imaging (MRI) revealed a strong gradient correlation between lactate and carotid wall thickness [77]. Consistently, a recent study has shown that lysine lactylation is markedly increased in atherosclerotic plaque regions compared with normal regions, which is closely associated with local shear stress patterns [78].

Endothelial-to-mesenchymal transition (EndMT) is a key driver contributing to vascular remodeling and plaque development in atherosclerosis [79]. Previous studies have shown that lactate promotes EndMT [80,81]. Dong et al. identified anti-silencing function 1A histone chaperone (ASF1A) as a cofactor for p300 [82]. p300 precisely regulates the enrichment of H3K18la at the promoter of snail family transcriptional repressor 1 (SNAI1), thereby activating SNAI1 transcription and promoting EndMT.

VSMCs proliferation and phenotypic switching represent key events in plaque formation and progression [83]. Zhu et al. found that MCT3 mRNA and protein expression correlate with AS severity, and impaired lactate transport caused by MCT3 inhibition may promote VSMC proliferation and induce AS [84]. In addition, Li et al. demonstrated that TRAP1-mediated metabolic reprogramming promotes histone H4 lysine 12 lactylation (H4K12la) through the downregulation of HDAC3 [85]. Increased H4K12la enriches promoters of senescence-associated secretory phenotype (SASP) genes, enhancing their transcription, accelerating VSMC senescence, and thereby promoting atherosclerosis. Furthermore, Xu et al. showed that persistent inflammatory injury induces TNFα-dependent SOX10 lactylation through activation of the PI3K/AKT signaling pathway, leading to VSMC transdifferentiation [86]. This process results in the accumulation of macrophage-like VSMCs, enhanced vascular inflammation, pyroptosis-driven hyperplasia, and the progression of atherosclerosis-associated complications.

In addition to ECs and VSMCs, macrophages are a critical component of the atherosclerotic plaque microenvironment [87], where lactylation may play a central regulatory role. Histone lactylation, first identified in macrophages during inflammatory responses, links glycolytic metabolism to gene expression and is particularly relevant in plaques characterized by hypoxia and high lactate levels. In this setting, lactate-driven lactylation (e.g., H3K18la) modulates macrophage gene programs, influencing polarization and function [88]. In some contexts, lactylation promotes reparative gene expression (e.g., *arginase 1*, *transforming growth factor-β1*, *interleukin-10*). However, its effects in atherosclerosis are complex and context-dependent, supporting both pro-inflammatory and pro-fibrotic roles depending on lesion stage and local cues [89]. In addition to histones, lactylation of non-histone proteins such as pyruvate kinase M2 (PKM2) may further regulate macrophage metabolism and inflammatory signaling [90]. Together, these findings highlight macrophage lactylation as a dynamic and context-dependent regulator of plaque progression and stability, and a potential target for therapeutic intervention.

### 5.2. Pulmonary Hypertension

A key feature of pulmonary arterial hypertension (PAH) is the remodeling of small arteries driven by persistently elevated blood pressure [91,92,93], often accompanied by extracellular matrix remodeling and VSMCs proliferation. These changes thicken pulmonary arterioles and restrict blood flow. A growing body of evidence indicates that lactate and lactylation play important roles in PAH pathogenesis [94,95]. Kovacs et al. showed that PFKFB3 upregulation drives increased aerobic glycolysis, and the elevated lactate can induce extracellular signal-regulated kinase 1/2 (ERK1/2) phosphorylation and calpain activation, leading to collagen synthesis and proliferation of pulmonary arterial VSMCs in PAH [96]. Another key feature of PAH is pulmonary vasoconstriction. Jones et al. proposed that activation of lactate receptor GPR81 can enhance endothelin-1 (ET-1) synthesis in arterial VSMCs [48]. ET-1 binds to endothelin-A receptors, inducing vasoconstriction. Therefore, the glycolytic pathway and lactate signaling may become potential targets for PAH treatment.

In PAH, increased mitochondrial reactive oxygen species (mROS) and glycolysis have been confirmed. Chen et al. reported that a mROS-mediated glycolytic shift drives histone lactylation, thereby promoting hypoxic PAH [42]. Mechanistically, hypoxia increases mROS, inhibits HIF-1α hydroxylation, and activates the HIF-1α downstream signaling pathway. These induce glycolysis in VSMCs and promote lactate accumulation and histone lactylation. ChIP-seq analysis of H3K18la and HIF-1α found that HIF-1α targets bone morphogenetic protein 5 (BMP5) within PASMCs. Lactylation was observed at H3K18 and H4K5 sites of transient receptor potential cation channel subfamily C member 5 (TRPC5) and KIT proto-oncogene, receptor tyrosine kinase (KIT). Pharmacological intervention with an LDH inhibitor reduced histone lactylation and improved PASMC proliferation and vascular remodeling in hypoxic PAH rats. Future PAH treatment strategies should focus on regulating lactate metabolism and lactylation modification, aiming to slow disease progression by intervening in metabolic-epigenetic pathways.

### 5.3. Myocardial Infarction

Following myocardial infarction (MI), ischemia and tissue necrosis trigger a robust inflammatory response and vascular remodeling within the infarct and border zones [97]. Inflammatory macrophages, activated fibroblasts, and hypoxic cardiomyocytes exhibit elevated glycolysis and lactate production, leading to widespread metabolic reprogramming [98]. Acute myocardial infarction (AMI) is one of the most severe coronary artery diseases caused by coronary stenosis and occlusion, leading to ischemic necrosis of the myocardium. Coronary occlusion causes a sharp reduction in oxygen delivery to cardiomyocytes, leading to decreased mitochondrial oxidative phosphorylation and increased anaerobic glycolysis rates in cardiomyocytes. This naturally results in increased lactate production by cardiomyocytes, consistent with numerous clinical studies finding elevated circulating lactate levels in AMI patients [99]. Simultaneously, elevated circulating lactate levels are associated with patient prognosis. A study involving 1176 ST-segment elevation myocardial infarction (STEMI) patients found that increased lactate levels were associated with higher acute mortality and increased 30-day mortality [100]. Wu et al. revealed that normalized lactate load predicts in-hospital mortality and prognosis in AMI patients, with predictive performance improving at higher lactate levels [101]. This suggests that massive lactate accumulation can cause local tissue acidosis, further exacerbating cardiomyocyte damage. Fan et al. revealed the relationship after MI [80]. The acidic environment not only disrupts cell membrane integrity but also inhibits cardiomyocyte energy metabolism, leading to apoptosis and necrosis. This metabolic imbalance is an important injury mechanism in the early stages of myocardial infarction.

### 5.4. Heart Failure

In heart failure (HF), chronic metabolic stress leads to mitochondrial dysfunction, sustained glycolytic shift, and persistent lactate accumulation in cardiac and vascular tissues [102,103]. This metabolic reprogramming, coupled with epigenetic alterations such as lactylation, contributes to endothelial dysfunction, capillary rarefaction, and maladaptive ventricular remodeling. In recent years, the role of lactate in HF has been extensively explored in both basic and clinical research [104]. Studies indicate that increased blood lactate levels may predict poor prognosis in HF patients [105]. During acute heart failure (AHF), several mechanisms can alter the lactate production/clearance balance, promoting lactate accumulation. These include peripheral hypoperfusion (from low cardiac output, high central venous pressure, or vasoconstriction), sympathetic activation, hypoxemia, anemia, and hepatic or renal dysfunction. A recent study demonstrated that histone lactylation promotes pressure overload-induced cardiac hypertrophy and heart failure by regulating transforming growth factor-β2 (TGF-β2) expression [106]. Increased histone lactylation enhances the transcription of *TGF-β2*, thereby activating pro-fibrotic and hypertrophic signaling pathways that contribute to pathological cardiac remodeling and the progression to heart failure. Some of these mechanisms may collectively impair oxygen delivery to peripheral organs, accelerate disease progression, and worsen prognosis. HF patients often exhibit metabolic disorders, particularly significant alterations in cardiomyocyte energy metabolism. Metabolic reprogramming in HF gradually shifts cardiomyocytes towards anaerobic metabolism. Lactate, as a glycolytic metabolite, accumulates substantially in HF, reflecting the state of inadequate oxygen and energy supply to cardiomyocytes. Therefore, early lactate assessment in AHF patients helps identify at-risk individuals for more comprehensive treatment to improve outcomes [107]. Door-to-lactate clearance, the rate at which elevated lactate levels normalize with treatment, has emerged as a prognostic indicator in HF [108]. Impaired lactate clearance reflects persistent tissue hypoperfusion and metabolic dysfunction, identifying patients requiring more aggressive intervention.

### 5.5. Diabetic Vascular Complications

Diabetes is a major risk factor for CVD, as chronic hyperglycemia and insulin insufficiency or resistance induce endothelial dysfunction, inflammation, and oxidative stress [109,110], leading to reduced blood flow to the extremities [111]. Elevated fasting lactate levels have been observed in patients with obesity and type 2 diabetes, and increased circulating lactate is positively associated with insulin resistance [112,113,114]. Emerging evidence indicates that hyperglycemia-driven metabolic reprogramming elevates overall lactate and lactylation levels, with dysregulation of this axis now recognized as a unified mechanism driving diabetic vascular complications [115]. Histone lactylation (e.g., H3K14la) and non-histone lactylation (e.g., pyruvate kinase M2 and mitochondrial fission protein 1) have been shown to promote inflammatory responses and metabolic dysregulation in diabetic vascular complications [116,117]. These findings highlight the emerging role of the lactate–lactylation axis as a unifying mechanism and suggest new therapeutic targets for diabetic vascular complications.

### 5.6. Biomarker Versus Causal Mediator in Cardiovascular Disease

A critical question is whether lactate serves primarily as a biomarker of metabolic stress or as a direct driver of disease pathogenesis. Evidence supports a dual role [118]. Clinically, elevated circulating lactate correlates with disease severity and prognosis in myocardial infarction, heart failure, and cardiogenic shock, reflecting tissue hypoperfusion and metabolic dysfunction [119,120,121]. However, accumulating mechanistic studies demonstrate that lactate actively contributes to disease progression by modulating signaling pathways and inducing epigenetic reprogramming through lactylation. This promotes inflammation, vascular remodeling, endothelial-to-mesenchymal transition, and smooth muscle cell proliferation. Consistent with this, targeting lactate production, transport, or lactylation can attenuate disease phenotypes in preclinical models. Thus, lactate functions as both a biomarker and a context-dependent mediator of cardiovascular disease, with its relative impact determined by disease context, the magnitude and duration of lactate elevation, and the cell types involved.

## 6. Conclusions

Over the past several decades, our understanding of lactate has evolved dramatically from its original classification as a metabolic waste product to its current recognition as a central regulator of metabolism, signaling, and epigenetic regulation. Notably, accumulating evidence over the past five years has identified histone lactylation as a key mechanism linking cellular metabolic state to gene expression, with important implications for cardiovascular physiology and disease [122].

Despite these advances, several important questions remain unanswered. First, the full complement of enzymes responsible for mediating lactylation (lactyltransferases) and delactylation (delactylases), along with their substrate specificities and context-dependent regulation, remains incompletely defined. Second, the spatiotemporal dynamics of lactate metabolism and lactylation across different vascular beds, cell types, and stages of disease progression are still poorly understood. Third, how lactate–lactylation signaling integrates with other metabolic and epigenetic pathways, such as acetylation, methylation, succinylation, and redox signaling, requires further investigation.

To address these questions, future studies should leverage multi-omics approaches (transcriptomics, proteomics, metabolomics, epigenomics) to map lactate-related regulatory networks in both health and disease. Advanced imaging tools and single-cell technologies will also be essential for resolving cell-type-specific metabolic signatures and lactylation dynamics within complex tissues. Future studies should aim to dissect the spatiotemporal dynamics of lactate metabolism and lactylation within the cardiovascular system, with particular attention to how pathological stimuli, including hypoxia, shear stress, metabolic dysfunction, and chronic inflammation, influence these processes. Integrative multi-omics approaches, combining genomics, transcriptomics, proteomics, metabolomics, and epigenomics, will be essential for capturing the complexity and context-dependence of lactate-related signaling networks.

From a translational perspective, targeting the lactate–lactylation axis presents an exciting therapeutic frontier. Strategies such as modulating lactate production, inhibiting lactate transporters, or pharmacologically regulating lactylation enzymes may offer novel avenues for preventing or reversing cardiovascular damage. Furthermore, biomarkers derived from lactate metabolism and lactylation status could improve risk stratification, diagnosis, and monitoring of therapeutic efficacy in patients with cardiovascular diseases.

In summary, the redefinition of lactate from a metabolic waste product to a multifaceted regulator of cardiovascular biology underscores its potential as both a biomarker and a therapeutic target. Continued exploration of the functional roles of lactate and lactylation will not only deepen our understanding of cardiovascular disease pathogenesis but also catalyze the development of next-generation precision therapies.

## Data Availability

No new data were created or analyzed in this study. Data sharing is not applicable.

## Abbreviations

AARS1	Alanyl-tRNA synthetase 1
ATP	adenosine triphosphate
AMP	adenosine monophosphate
AMPK	AMP-activated protein kinase
BMP	bone morphogenetic protein
CAD	coronary artery disease
CVD	cardiovascular disease
DHAP	dihydroxyacetone phosphate
EC	endothelial cell
ECM	extracellular matrix
EndMT	endothelial-to-mesenchymal transition
GLUT	glucose transporter
GPR	G-protein-coupled receptor
HDAC	histone deacetylase
KATs	lysine acyltransferases
LDH	lactate dehydrogenase
MCTs	monocarboxylate transporters
MMP	matrix metalloproteinase
NADH	nicotinamide adenine dinucleotide
NO	nitric oxide
PAH	pulmonary arterial hypertension
PDH	pyruvate dehydrogenase
PTM	post-translational modification
TNFα	tumor necrosis factor-alpha
TCA	tricarboxylic acid
ROS	reactive oxygen species
VSMC	vascular smooth muscle cells
